# 3-Ethyl­sulfinyl-5-fluoro-2-(4-iodo­phen­yl)-1-benzofuran

**DOI:** 10.1107/S1600536810024736

**Published:** 2010-06-30

**Authors:** Hong Dae Choi, Pil Ja Seo, Byeng Wha Son, Uk Lee

**Affiliations:** a Department of Chemistry, Dongeui University, San 24 Kaya-dong Busanjin-gu, Busan 614-714, Republic of Korea; b Department of Chemistry, Pukyong National University, 599-1 Daeyeon 3-dong, Nam-gu, Busan 608-737, Republic of Korea

## Abstract

In the title compound, C_16_H_12_FIO_2_S, the 4-iodo­phenyl ring is rotated slightly out of the benzofuran plane, as indicated by the dihedral angle of 11.41 (7)°. The crystal structure is stabilized by an inter­molecular π–π inter­action between the benzene and 4-iodo­phenyl rings [centroid–centroid distance = 3.757 (3) Å]. The crystal structure also exhibits a weak inter­molecular C—H⋯O hydrogen bond and a short I⋯O [3.2575 (16) Å] contact.

## Related literature

For the pharmacological activity of benzofuran compounds, see: Aslam *et al.* (2006 ▶); Galal *et al.* (2009 ▶); Khan *et al.* (2005 ▶). For natural products with benzofuran rings, see: Akgul & Anil (2003 ▶); Soekamto *et al.* (2003 ▶). For structures of related 3-ethyl­sulfinyl-2-(4-fluoro­phen­yl)-5-halo-1-benzofuran derivatives, see: Choi *et al.* (2010**a* ▶,*b* ▶,c* ▶). For a review of halogen bonding, see: Politzer *et al.* (2007 ▶).
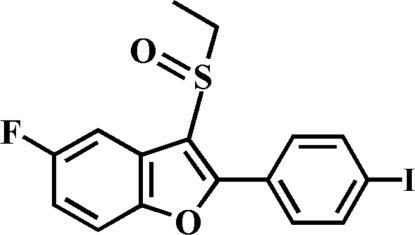


## Experimental

### 

#### Crystal data

C_16_H_12_FIO_2_S
*M* *_r_* = 414.22
Monoclinic, 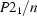

*a* = 7.5384 (4) Å
*b* = 9.4104 (5) Å
*c* = 20.3049 (9) Å
β = 96.298 (2)°
*V* = 1431.72 (12) Å^3^
*Z* = 4
Mo *K*α radiation
μ = 2.39 mm^−1^
*T* = 173 K
0.24 × 0.18 × 0.16 mm

#### Data collection

Bruker SMART APEXII CCD diffractometer
Absorption correction: multi-scan (*SADABS*; Bruker, 2009 ▶) *T*_min_ = 0.658, *T*_max_ = 0.680
12915 measured reflections
3265 independent reflections
3061 reflections with *I* > 2σ(*I*)
*R*_int_ = 0.030

#### Refinement

*R*[*F* ^2^ > 2σ(*F* ^2^)] = 0.024
*wR*(*F* ^2^) = 0.062
*S* = 1.13
3265 reflections
191 parameters
H-atom parameters constrained
Δρ_max_ = 0.53 e Å^−3^
Δρ_min_ = −0.78 e Å^−3^

### 

Data collection: *APEX2* (Bruker, 2009 ▶); cell refinement: *SAINT* (Bruker, 2009 ▶); data reduction: *SAINT*; program(s) used to solve structure: *SHELXS97* (Sheldrick, 2008 ▶); program(s) used to refine structure: *SHELXL97* (Sheldrick, 2008 ▶); molecular graphics: *ORTEP-3* (Farrugia, 1997 ▶) and *DIAMOND* (Brandenburg, 1998 ▶); software used to prepare material for publication: *SHELXL97*.

## Supplementary Material

Crystal structure: contains datablocks global, I. DOI: 10.1107/S1600536810024736/is2566sup1.cif

Structure factors: contains datablocks I. DOI: 10.1107/S1600536810024736/is2566Isup2.hkl

Additional supplementary materials:  crystallographic information; 3D view; checkCIF report

## Figures and Tables

**Table 1:** Hydrogen-bond geometry (Å, °)

*D*—H⋯*A*	*D*—H	H⋯*A*	*D*⋯*A*	*D*—H⋯*A*
C9—H9*B*⋯O2^i^	0.97	2.59	3.337 (3)	134

Symmetry code: (i) 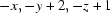.

**Fig. 1.**
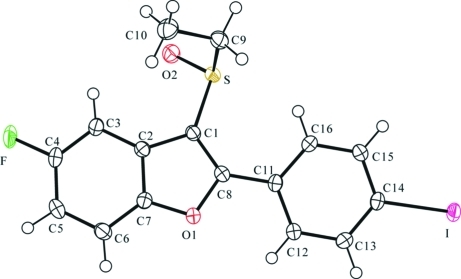
The molecular structure of the title compound with the atom numbering scheme. Displacement ellipsoids are drawn at the 50% probability level. H atoms are presented as a small spheres of arbitrary radius.

**Fig. 2.**
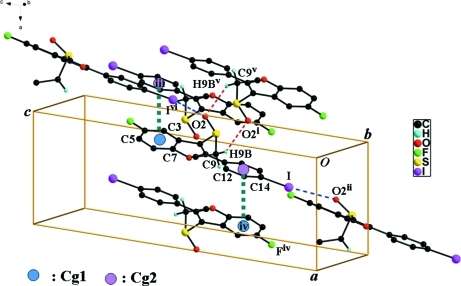
π–π, C—H···O, and I···O interactions (dotted lines) in the crystal structure of the title compound. Cg1 and Cg2 denote the ring centroids. [Symmetry codes: (i) - x, - y + 2, - z + 1; (ii) x + 1/2, - y + 1/2, z - 1/2; (iii) - x, - y +1, - z + 1; (iv) - x + 1, - y + 1, - z + 1; (v) x - 1/2, - y + 3/2, z + 1/2.]

## supplementary crystallographic information

### Comment

Compounds involving a benzofuran moiety show potent pharmacological properties such as antifungal (Aslam *et al.*, 2006), antitumor and antiviral (Galal *et al.*,  2009), antimicrobial (Khan *et al.*, 2005) activities. These compounds occur widely in nature (Akgul & Anil, 2003; Soekamto *et al.*, 2003). As a part of our ongoing studies of the substituent effect on the solid state structures of 3-ethylsulfinyl-2-(4-fluorophenyl)-5-halo-1-benzofuran analogues (Choi *et al.*, 2010*a,b,c*), we report the crystal structure of the title compound (Fig. 1).

The benzofuran unit is essentially planar, with a mean deviation of 0.011 (2)Å from the least-squares plane defined by the nine constituent atoms. The dihedral angle formed by the benzofuran plane and the 4-iodophenyl ring is 11.41 (7)°. The crystal packing (Fig. 2) is stabilized by aromatic π–π interactions between the benzene and the 4-iodophenyl rings of the neighbouring molecules, with a *Cg*1···*Cg*2^iii^ distance of 3.757 (3) Å (*Cg*1 and *Cg*2 are the centroids of the C2–C7 benzene ring and the C11–C16 4-iodophenyl ring, respectively). The molecular packing (Fig. 2) is further stabilized by a weak intermolecular C—H···O hydrogen bond between the methylene H atom of the ethyl group and the oxygen of the S═O unit, with a C9—H9B···O2^i^ (Table 1). The crystal packing (Fig. 2) also exhibits an I···O halogen bonding between the iodine and the oxygen of the S═O unit [I···O2^ii^ = 3.2575 (16) Å; C14—I···O2^ii^ = 153.53 (6)°] (Politzer *et al.*, 2007).

### Experimental

77% 3-Chloroperoxybenzoic acid (202 mg, 0.9 mmol) was added in small portions to a stirred solution of 3-ethylsulfanyl-5-fluoro-2-(4-iodophenyl)-1-benzofuran (318 mg, 0.8 mmol) in dichloromethane (40 mL) at 273 K. After being stirred at room temperature for 4h, the mixture was washed with saturated sodium bicarbonate solution and the organic layer was separated, dried over magnesium sulfate, filtered and concentrated at reduced pressure. The residue was purified by column chromatography (hexane–ethyl acetate, 1:1 *v*/*v*) to afford the title compound as a colorless solid [yield 78%, m.p. 434–435 K; *R*_f_ = 0.59 (hexane-ethyl acetate, 1:1 *v*/*v*)]. Single crystals suitable for X-ray diffraction were prepared by slow evaporation of a solution of the title compound in tetrahydrofuran at room temperature.

### Refinement

All H atoms were positioned geometrically and refined using a riding model, with C—H = 0.93 Å for aryl, 0.97 Å for methylene, and 0.96 Å for methyl H atoms, and with *U*_iso_(H) = 1.2*U*_eq_(C) for aryl and methylene H atoms, and 1.5*U*_eq_(C) for methyl H atoms.

### Crystal data

**Table:**

C_16_H_12_FIO_2_S	*F*(000) = 808
*M**_r_* = 414.22	*D*_x_ = 1.922 Mg m^−^^3^
Monoclinic, *P*2_1_/*n*	Mo *K*α radiation, λ = 0.71073 Å
Hall symbol: -P 2yn	Cell parameters from 8330 reflections
*a* = 7.5384 (4) Å	θ = 2.4–27.5°
*b* = 9.4104 (5) Å	µ = 2.39 mm^−^^1^
*c* = 20.3049 (9) Å	*T* = 173 K
β = 96.298 (2)°	Block, colourless
*V* = 1431.72 (12) Å^3^	0.24 × 0.18 × 0.16 mm
*Z* = 4	

### Data collection

**Table:**

Bruker SMART APEXII CCD diffractometer	3265 independent reflections
Radiation source: rotating anode	3061 reflections with *I* > 2σ(*I*)
graphite multilayer	*R*_int_ = 0.030
Detector resolution: 10.0 pixels mm^-1^	θ_max_ = 27.5°, θ_min_ = 2.0°
φ and ω scans	*h* = −9→9
Absorption correction: multi-scan (*SADABS*; Bruker, 2009)	*k* = −11→12
*T*_min_ = 0.658, *T*_max_ = 0.680	*l* = −26→26
12915 measured reflections	

### Refinement

**Table:**

Refinement on *F*^2^	Primary atom site location: structure-invariant direct methods
Least-squares matrix: full	Secondary atom site location: difference Fourier map
*R*[*F*^2^ > 2σ(*F*^2^)] = 0.024	Hydrogen site location: difference Fourier map
*wR*(*F*^2^) = 0.062	H-atom parameters constrained
*S* = 1.13	*w* = 1/[σ^2^(*F*_o_^2^) + (0.0234*P*)^2^ + 1.1834*P*] where *P* = (*F*_o_^2^ + 2*F*_c_^2^)/3
3265 reflections	(Δ/σ)_max_ = 0.001
191 parameters	Δρ_max_ = 0.53 e Å^−^^3^
0 restraints	Δρ_min_ = −0.78 e Å^−^^3^

### Special details

**Table:**

Geometry. All esds (except the esd in the dihedral angle between two l.s. planes) are estimated using the full covariance matrix. The cell esds are taken into account individually in the estimation of esds in distances, angles and torsion angles; correlations between esds in cell parameters are only used when they are defined by crystal symmetry. An approximate (isotropic) treatment of cell esds is used for estimating esds involving l.s. planes.
Refinement. Refinement of F^2^ against ALL reflections. The weighted R-factor wR and goodness of fit S are based on F^2^, conventional R-factors R are based on F, with F set to zero for negative F^2^. The threshold expression of F^2^ > 2sigma(F^2^) is used only for calculating R-factors(gt) etc. and is not relevant to the choice of reflections for refinement. R-factors based on F^2^ are statistically about twice as large as those based on F, and R- factors based on ALL data will be even larger.

### Fractional atomic coordinates and isotropic or equivalent isotropic displacement parameters (Å^2^)

**Table:**

	*x*	*y*	*z*	*U*_iso_*/*U*_eq_	
I	0.410489 (19)	0.499975 (13)	0.189463 (7)	0.02345 (7)	
S	0.10698 (7)	0.80859 (5)	0.50166 (2)	0.01980 (11)	
F	0.07034 (19)	0.56507 (16)	0.76127 (6)	0.0317 (3)	
O1	0.27247 (19)	0.40664 (15)	0.52431 (7)	0.0203 (3)	
O2	−0.0186 (2)	0.85501 (17)	0.54905 (8)	0.0283 (3)	
C1	0.1851 (3)	0.6367 (2)	0.52771 (10)	0.0183 (4)	
C2	0.1707 (3)	0.5743 (2)	0.59203 (10)	0.0184 (4)	
C3	0.1180 (3)	0.6214 (2)	0.65229 (10)	0.0210 (4)	
H3	0.0824	0.7147	0.6583	0.025*	
C4	0.1218 (3)	0.5224 (2)	0.70199 (11)	0.0227 (4)	
C5	0.1728 (3)	0.3806 (2)	0.69609 (11)	0.0246 (4)	
H5	0.1710	0.3185	0.7316	0.030*	
C6	0.2257 (3)	0.3339 (2)	0.63685 (11)	0.0234 (4)	
H6	0.2605	0.2403	0.6311	0.028*	
C7	0.2246 (3)	0.4326 (2)	0.58651 (10)	0.0191 (4)	
C8	0.2457 (3)	0.5318 (2)	0.48905 (10)	0.0181 (4)	
C9	0.3111 (3)	0.9094 (2)	0.52147 (11)	0.0242 (4)	
H9A	0.4062	0.8643	0.5006	0.029*	
H9B	0.2944	1.0041	0.5030	0.029*	
C10	0.3674 (3)	0.9214 (3)	0.59512 (11)	0.0296 (5)	
H10A	0.2726	0.9633	0.6164	0.044*	
H10B	0.4720	0.9799	0.6025	0.044*	
H10C	0.3934	0.8285	0.6132	0.044*	
C11	0.2883 (3)	0.5241 (2)	0.42083 (11)	0.0185 (4)	
C12	0.3152 (3)	0.3921 (2)	0.39142 (10)	0.0215 (4)	
H12	0.3078	0.3092	0.4159	0.026*	
C13	0.3528 (3)	0.3837 (2)	0.32607 (11)	0.0229 (4)	
H13	0.3720	0.2957	0.3072	0.028*	
C14	0.3616 (3)	0.5066 (2)	0.28903 (11)	0.0199 (4)	
C15	0.3361 (3)	0.6385 (2)	0.31721 (11)	0.0233 (4)	
H15	0.3432	0.7209	0.2923	0.028*	
C16	0.3001 (3)	0.6469 (2)	0.38255 (11)	0.0234 (4)	
H16	0.2834	0.7354	0.4013	0.028*	

### Atomic displacement parameters (Å^2^)

**Table:**

	*U*^11^	*U*^22^	*U*^33^	*U*^12^	*U*^13^	*U*^23^
I	0.02965 (10)	0.02541 (10)	0.01641 (9)	−0.00231 (5)	0.00756 (6)	−0.00183 (4)
S	0.0218 (2)	0.0183 (2)	0.0192 (2)	0.00129 (18)	0.00201 (18)	0.00248 (18)
F	0.0415 (8)	0.0402 (8)	0.0148 (6)	0.0028 (6)	0.0099 (5)	0.0013 (5)
O1	0.0263 (7)	0.0193 (7)	0.0158 (7)	0.0016 (6)	0.0041 (6)	0.0012 (5)
O2	0.0297 (8)	0.0282 (8)	0.0282 (8)	0.0049 (7)	0.0091 (7)	0.0018 (7)
C1	0.0197 (9)	0.0178 (9)	0.0173 (9)	0.0000 (7)	0.0016 (7)	0.0018 (7)
C2	0.0177 (9)	0.0188 (10)	0.0184 (9)	−0.0002 (7)	0.0012 (7)	0.0009 (8)
C3	0.0220 (10)	0.0239 (10)	0.0173 (9)	0.0010 (8)	0.0034 (8)	−0.0009 (8)
C4	0.0232 (10)	0.0305 (11)	0.0149 (10)	−0.0011 (8)	0.0050 (8)	−0.0007 (8)
C5	0.0273 (10)	0.0264 (11)	0.0200 (10)	−0.0012 (9)	0.0022 (8)	0.0078 (8)
C6	0.0264 (10)	0.0206 (10)	0.0230 (10)	0.0005 (8)	0.0018 (8)	0.0033 (8)
C7	0.0196 (9)	0.0204 (10)	0.0175 (9)	−0.0008 (8)	0.0026 (8)	−0.0007 (8)
C8	0.0177 (9)	0.0187 (9)	0.0177 (10)	−0.0010 (7)	0.0010 (8)	0.0023 (8)
C9	0.0254 (10)	0.0225 (10)	0.0252 (11)	−0.0049 (8)	0.0048 (9)	0.0014 (8)
C10	0.0294 (11)	0.0307 (12)	0.0283 (12)	−0.0051 (9)	0.0015 (9)	−0.0041 (9)
C11	0.0165 (9)	0.0230 (10)	0.0163 (10)	0.0000 (7)	0.0026 (7)	−0.0002 (8)
C12	0.0264 (10)	0.0209 (10)	0.0179 (10)	0.0010 (8)	0.0055 (8)	0.0017 (8)
C13	0.0278 (10)	0.0201 (10)	0.0211 (10)	0.0022 (8)	0.0042 (8)	−0.0021 (8)
C14	0.0206 (10)	0.0249 (11)	0.0144 (10)	−0.0008 (7)	0.0026 (8)	−0.0017 (7)
C15	0.0314 (11)	0.0201 (10)	0.0189 (10)	−0.0003 (8)	0.0050 (8)	0.0030 (8)
C16	0.0321 (11)	0.0199 (10)	0.0192 (10)	0.0009 (8)	0.0067 (8)	−0.0023 (8)

### Geometric parameters (Å, °)

**Table:**

I—C14	2.095 (2)	C6—H6	0.9300
I—O2^i^	3.2575 (16)	C8—C11	1.458 (3)
S—O2	1.4876 (17)	C9—C10	1.513 (3)
S—C1	1.782 (2)	C9—H9A	0.9700
S—C9	1.815 (2)	C9—H9B	0.9700
F—C4	1.365 (3)	C10—H10A	0.9600
O1—C7	1.373 (2)	C10—H10B	0.9600
O1—C8	1.381 (2)	C10—H10C	0.9600
C1—C8	1.370 (3)	C11—C16	1.401 (3)
C1—C2	1.447 (3)	C11—C12	1.403 (3)
C2—C3	1.399 (3)	C12—C13	1.389 (3)
C2—C7	1.402 (3)	C12—H12	0.9300
C3—C4	1.372 (3)	C13—C14	1.385 (3)
C3—H3	0.9300	C13—H13	0.9300
C4—C5	1.397 (3)	C14—C15	1.389 (3)
C5—C6	1.380 (3)	C15—C16	1.385 (3)
C5—H5	0.9300	C15—H15	0.9300
C6—C7	1.380 (3)	C16—H16	0.9300
			
C14—I—O2^i^	153.53 (6)	C10—C9—H9A	108.9
O2—S—C1	106.72 (9)	S—C9—H9A	108.9
O2—S—C9	106.71 (10)	C10—C9—H9B	108.9
C1—S—C9	99.45 (10)	S—C9—H9B	108.9
C7—O1—C8	106.66 (15)	H9A—C9—H9B	107.7
C8—C1—C2	106.71 (18)	C9—C10—H10A	109.5
C8—C1—S	127.16 (16)	C9—C10—H10B	109.5
C2—C1—S	125.55 (15)	H10A—C10—H10B	109.5
C3—C2—C7	118.95 (18)	C9—C10—H10C	109.5
C3—C2—C1	135.82 (19)	H10A—C10—H10C	109.5
C7—C2—C1	105.23 (17)	H10B—C10—H10C	109.5
C4—C3—C2	116.3 (2)	C16—C11—C12	118.3 (2)
C4—C3—H3	121.9	C16—C11—C8	121.35 (19)
C2—C3—H3	121.9	C12—C11—C8	120.38 (19)
F—C4—C3	117.6 (2)	C13—C12—C11	120.73 (19)
F—C4—C5	117.6 (2)	C13—C12—H12	119.6
C3—C4—C5	124.7 (2)	C11—C12—H12	119.6
C6—C5—C4	119.2 (2)	C14—C13—C12	119.9 (2)
C6—C5—H5	120.4	C14—C13—H13	120.1
C4—C5—H5	120.4	C12—C13—H13	120.1
C5—C6—C7	116.9 (2)	C13—C14—C15	120.4 (2)
C5—C6—H6	121.5	C13—C14—I	121.53 (15)
C7—C6—H6	121.5	C15—C14—I	118.10 (15)
O1—C7—C6	125.48 (19)	C16—C15—C14	119.7 (2)
O1—C7—C2	110.53 (17)	C16—C15—H15	120.1
C6—C7—C2	123.98 (19)	C14—C15—H15	120.1
C1—C8—O1	110.86 (18)	C15—C16—C11	121.0 (2)
C1—C8—C11	134.61 (19)	C15—C16—H16	119.5
O1—C8—C11	114.53 (18)	C11—C16—H16	119.5
C10—C9—S	113.40 (16)		
			
O2—S—C1—C8	153.69 (18)	C2—C1—C8—O1	−0.4 (2)
C9—S—C1—C8	−95.6 (2)	S—C1—C8—O1	−171.94 (14)
O2—S—C1—C2	−16.3 (2)	C2—C1—C8—C11	−179.9 (2)
C9—S—C1—C2	94.44 (18)	S—C1—C8—C11	8.6 (4)
C8—C1—C2—C3	−179.7 (2)	C7—O1—C8—C1	1.0 (2)
S—C1—C2—C3	−8.0 (3)	C7—O1—C8—C11	−179.43 (17)
C8—C1—C2—C7	−0.3 (2)	O2—S—C9—C10	42.74 (19)
S—C1—C2—C7	171.38 (15)	C1—S—C9—C10	−68.02 (18)
C7—C2—C3—C4	−0.6 (3)	C1—C8—C11—C16	11.3 (4)
C1—C2—C3—C4	178.7 (2)	O1—C8—C11—C16	−168.10 (19)
C2—C3—C4—F	−179.70 (18)	C1—C8—C11—C12	−167.4 (2)
C2—C3—C4—C5	−0.4 (3)	O1—C8—C11—C12	13.2 (3)
F—C4—C5—C6	−179.96 (19)	C16—C11—C12—C13	0.1 (3)
C3—C4—C5—C6	0.8 (4)	C8—C11—C12—C13	178.88 (19)
C4—C5—C6—C7	0.0 (3)	C11—C12—C13—C14	−0.9 (3)
C8—O1—C7—C6	177.8 (2)	C12—C13—C14—C15	1.1 (3)
C8—O1—C7—C2	−1.2 (2)	C12—C13—C14—I	−178.22 (16)
C5—C6—C7—O1	−179.99 (19)	C13—C14—C15—C16	−0.6 (4)
C5—C6—C7—C2	−1.1 (3)	I—C14—C15—C16	178.77 (17)
C3—C2—C7—O1	−179.52 (17)	C14—C15—C16—C11	−0.2 (3)
C1—C2—C7—O1	1.0 (2)	C12—C11—C16—C15	0.4 (3)
C3—C2—C7—C6	1.4 (3)	C8—C11—C16—C15	−178.3 (2)
C1—C2—C7—C6	−178.09 (19)		

Symmetry codes: (i) *x*+1/2, −*y*+3/2, *z*−1/2.

### Hydrogen-bond geometry (Å, °)

**Table:**

*D*—H···*A*	*D*—H	H···*A*	*D*···*A*	*D*—H···*A*
C9—H9B···O2^ii^	0.97	2.59	3.337 (3)	134

Symmetry codes: (ii) −*x*, −*y*+2, −*z*+1.
